# Sparse Unorganized Point Cloud Based Relative Pose Estimation for Uncooperative Space Target

**DOI:** 10.3390/s18041009

**Published:** 2018-03-28

**Authors:** Fang Yin, Wusheng Chou, Yun Wu, Guang Yang, Song Xu

**Affiliations:** 1 School of Mechanical Engineering and Automation, Beihang University, Beijing 100191, China; wschou@buaa.edu.cn (W.C.); yg_id@buaa.edu.cn (G.Y.); keithxs@buaa.edu.cn (S.X.); 2 State Key Laboratory of Virtual Reality Technology and Systems, Beihang University, Beijing 100191, China; 3 Beijing Institute of Control Engineering, Beijing 100080, China; wuy04110@163.com

**Keywords:** uncooperative target, congruent tetrahedron align, two-level index hash table, iterative closest point

## Abstract

This paper proposes an autonomous algorithm to determine the relative pose between the chaser spacecraft and the uncooperative space target, which is essential in advanced space applications, e.g., on-orbit serving missions. The proposed method, named Congruent Tetrahedron Align (CTA) algorithm, uses the very sparse unorganized 3D point cloud acquired by a LIDAR sensor, and does not require any prior pose information. The core of the method is to determine the relative pose by looking for the congruent tetrahedron in scanning point cloud and model point cloud on the basis of its known model. The two-level index hash table is built for speeding up the search speed. In addition, the Iterative Closest Point (ICP) algorithm is used for pose tracking after CTA. In order to evaluate the method in arbitrary initial attitude, a simulated system is presented. Specifically, the performance of the proposed method to provide the initial pose needed for the tracking algorithm is demonstrated, as well as their robustness against noise. Finally, a field experiment is conducted and the results demonstrated the effectiveness of the proposed method.

## 1. Introduction

On-Orbit Servicing (OOS), including on-orbit assembly, on-orbit maintenance and on-orbit fueling is an important developing technology for future space systems [1,2,3]. Furthermore, the accurate and reliable relative pose determination between chaser and target is one of the key techniques to accomplish the OOS mission successfully.

The relative pose determination research on cooperative targets [4,5,6], which are equipped with a set of artificial active or passive markers on the target surfaces that can be easily detected and recognized in the acquired datasets, has many achievements and some of them [7,8] have been successfully applied in orbit. However, it is more challenging to estimate the relative pose of the uncooperative targets without cooperative information, especially for the fast-moving targets, such as the high-speed tumbling uncooperative target. Therefore, the pose estimate accuracy and the computational cost have put forward higher requirements. According to the different electro-optical (EO) sensors on the chaser, the study of the uncooperative target is generally divided into the relative pose calculation based on passive systems (e.g., monocular and stereo system) and active systems (e.g., LIDAR). In addition, methods of multi-sensor information fusion are proposed [9,10], e.g., monocular and LIDAR. The methods based on the passive system tend to achieve better accuracy, but they are time-consuming and susceptible to illumination changes. However, the LIDAR system has stronger robustness for illumination.

This paper proposes a novel method of relative pose determination using the very sparse unorganized point cloud acquired by the scanning LIDAR sensor. We propose a Congruent Tetrahedron Align (CTA) method to calculate relative pose directly by searching congruent tetrahedron in scanning point cloud and model point cloud which is acquired from the known 3D CAD model of target. Moreover, this method does not need the time consumption process such as extracting feature [11] or calculating the normal which is a line perpendicular to the local surface represented by a point and its neighbors, and that does not need dense point cloud. The Iterative Closest Point (ICP) [12] algorithm which has advantages of high precision and high speed, can be used for pose tracking since the initial pose has been obtained.

The rest of this paper is organized as follows. In Section 2, the related works in recent years are described in detail. In Section 3, the details of proposed pose estimation method are presented. Then, we analyze the influencing factors of the proposed method in Section 4. Experiments and conclusions are shown in Section 5 and Section 6, respectively.

## 2. Related Work

The research on relative pose determination of uncooperative targets is a fundamental problem for OOS application. Because of their lower hardware complexity, their lower cost and their lower power consumption, passive systems have been widely considered in the research field of relative position calculation of space non-cooperative targets [13,14,15].

Methods based on monocular cameras that rely on various Perspective-n-Point problem (PnP) solvers [16,17,18] have been proposed to calculate the initial relative pose. Lepetit et al. [16] proposed efficient PnP (EPnP) solvers with the main idea of expressing the reference points as a weighted sum of four non-coplanar virtual control points. D’Amico [14] proposed the Newton-Raphson method to solve the PnP iteratively. Due to its use of least squares, which is very suitable for handling Gaussian noise, it has a high accuracy even in the presence of outliers or noise. In addition, the two-dimensional Template Matching (2D TM) approaches [19] are usually advanced in high accuracy and good anti-noise performance. Cai et al. [15] proposed a hollow annulus structure according to the Features from Accelerated Segment (FAST) feature to match the template and object image by comparing the differences of both grey and gradient. It is very important and challenging to obtain the correct correspondence between the features extracted from the image and the target model before approaching the PnP solvers. Besides the method based on point features, some others used the line features, which are typically more stable than points. For the stereo system, SoftPOSIT [20] that combines the iterative soft-assign algorithm [21,22] and the iterative POSIT algorithm [23] has achieved good accuracy. However, all the above methods generally take more than one second. Hence, when there are other celestial bodies in the field of view, it is not easy for the passive system to distinguish correctly, and the performance is obviously affected by the spatial illumination. In contrast, the active system of LIDAR system has the stronger robustness to the space illumination, and is easier to differentiate targets from the background. Therefore, the methods based on LIDAR are receiving more and more extensive research.

3D TM algorithm [24,25] evolves from the 2D TM algorithm we introduced above. In the process of 3D TM algorithm, a pose space database is generated from a set of views of the 3D target model. Then, the best initial pose estimate is defined as the pose that produces the best correlation with the measurement data. In order to get sufficient accuracy, this method needs to search the entire pose space, which also leads to high computational cost and limits its real-time spatial application. Hence, Opromolla et al., adopt an on-line 3D TM algorithm [26,27,28] to overcome this limitation. Specifically, the relative position is computed by the centroiding approach, thus, the amount of templates is reduced, and the database of templates can be cut down to 3-DOF. In order to further reduce the calculation time, the Principal Component Analysis (PCA) method is used to estimate the main axis, and the database is cut down to 1-DOF, which significantly reduces the searching time. However, the target is required to have an elongated shape.

Besides that, there are some feature-based methods. John O. Woods and John A. Christian proposed an Oriented, Unique, and Repeatable Clustered Viewpoint Feature Histograms (OUR-CVFH) algorithm [11,29], which has the same runtime complexity as ICP (in many cases it runs more quickly), provides free object recognition, and is robust to many types of occlusion. Specifically, a database consists of a set of 303-bin OUR-CVFH histograms describing that each segmented object is built off-line. The OUR-CVFH works when the LIDAR data are obtained with multiplicative EKF to provide a propagated pose estimate before refining the estimate using ICP. This feature-based method generally requires evenly distributed high resolution 3D data to calculate the normal of points.

In addition to the 3D TM methods and feature-based methods described above, the original point-based approaches have also been proposed for space applications. Liu et al. proposed a novel global point cloud registration algorithm based on the translation domain estimation method [30]. The translation domain is estimated by calculating the relative displacements of the axis-aligned bounding box (AABB) in the three main directions of the model point cloud and the scanning point cloud. Then, the GO-ICP algorithm [31], combined BnB (Branch-and-Bound) strategy [32] and ICP algorithm is used to search the optimal estimate. The Polygonal Aspect Hashing (PAH) algorithm [33] proposed by Ruel et al., provides 6-DOF relative pose information in real time based on the idea of puzzles, which aligns the model surface to one or more polygons extracted from scanning point cloud data.

The paper presents another point based method called CTA for the acquisition of the initial pose improved from PAH. The CTA algorithm finds the congruent tetrahedrons, which are from model point cloud and scan point cloud respectively. Some other algorithms for CTA are combined in the process. Specifically, 3D hull algorithm is used to construct a 3D convex from scanning point cloud data for selecting a tetrahedron, and a two-level index hash table is used to speed up the congruent tetrahedron searching speed.

## 3. Proposed Relative Pose Determination Method

**Figure 1:**
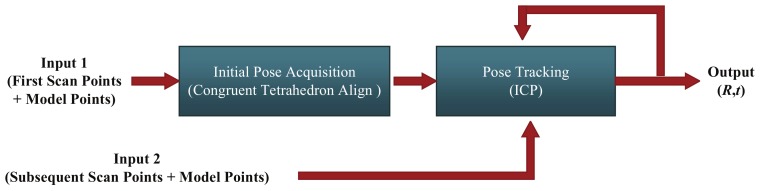
Logical scheme of the proposed method for relative pose determination.

The logical scheme of the process to determine relative pose of uncooperative target is shown in Figure 1. The process is divided into two main steps, named initial pose acquisition and pose tracking, respectively. The proposed CTA algorithm is applied in the initial pose acquisition process when the first scan point cloud is obtained from the scanning LIDAR. The model point cloud can be obtained from the 3D CAD model, which is known. Pose tracking is performed by means of ICP algorithm, which requires a good initial pose estimate. The details of the proposed method are described in the following parts of this section.

**Figure 2:**
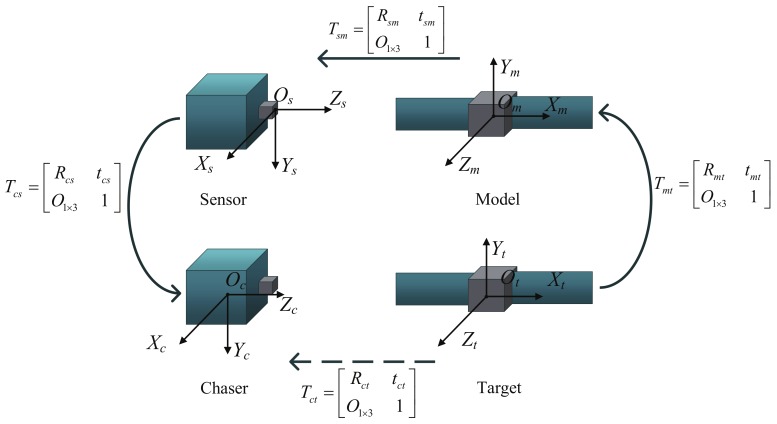
The definition of reference frames [30].

Relative pose determination is the problem of calculating the 3D rotation $R_{tc}$ and translation $t_{tc}$ between chaser and target (Figure 2). Furthermore, four reference frames are of interest: the chaser body-fixed frame *O*${}_{c}$*X*${}_{c}$*Y*${}_{c}$*Z*${}_{c}$, the sensor frame *O*${}_{s}$*X*${}_{s}$*Y*${}_{s}$*Z*${}_{s}$, the target model frame *O*${}_{m}$*X*${}_{m}$*Y*${}_{m}$*Z*${}_{m}$, and the target body-fixed frame *O*${}_{t}$*X*${}_{t}$*Y*${}_{t}$*Z*${}_{t}$.

$T_{ij}$ denotes the transformation (including rotation and translation) from frame *j* to frame *i*. Generally, $T_{cs}$ can be measured offline, depending on the assembly relation of chaser and sensor. $T_{mt}$ can be obtained from the definition of model frame. $T_{ct}$, the rotation and translation from target to chaser, can be calculated by Equation (1), where due to the achievable knowledge of $T_{cs}$ and $T_{mt}$, we only need to calculate $T_{sm}$.

(1) $$\begin{array}{c} T_{ct}=T_{cs}T_{sm}T_{mt} \end{array}$$

### 3.1. CTA Algorithm

**Figure 3:**
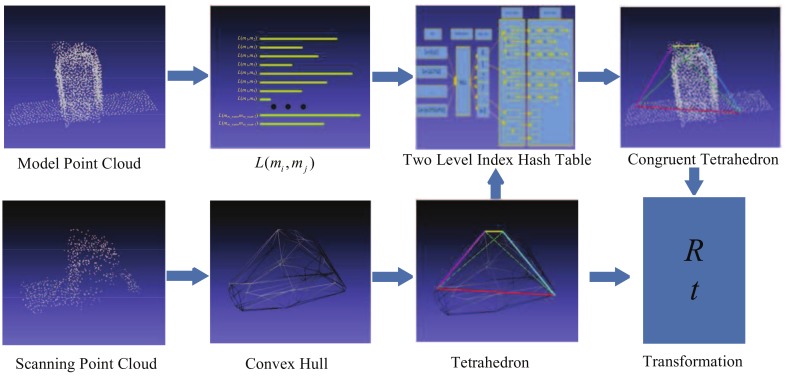
Flow of CTA Algorithm.

Essentially, the relative pose determination, or registration, consists of finding the overlap between the model point cloud and the scan point cloud, and then calculating the relative transformation. Instead of extracting features, our method finds the congruent tetrahedron directly from the scanning point cloud and model point cloud, and then estimates the relative pose by aligning the congruent tetrahedron. The procedure of the algorithm is illustrated in Figure 3. The model point cloud is sampled from the known 3D CAD model of target. Let $L(m_{i},m_{j})$ be the length of point pairs in the model point cloud. In order to search the corresponding point quickly and accurately, a two-level index hash table which has a low-time complexity of looking up is designed for storing the point pair length as well as the location topology information. When the scanning point cloud is obtained, a 3D convex hull will be constructed to simplify it. Then a tetrahedron $T_{s}$ with the largest volume is found in the vertices of the convex hull. According to the congruence theorem for tetrahedra, the corresponding tetrahedron of $T_{s}$ is found in the model point cloud, and then the transformation between them will be calculated.

#### 3.1.1. Congruent Tetrahedron Searching Based on Two-Level Index Hash Table

One of the necessary and sufficient conditions for two tetrahedrons to be congruent is as follows: If six sides of a tetrahedron are equal to the corresponding six sides of another tetrahedron, then the tetrahedrons are regarded as being congruent. Therefore, we find the congruent tetrahedrons, which are respectively from model point cloud and scanning point cloud according to the equal corresponding six sides. In order to find the corresponding sides conveniently and rapidly, we use the two-level index hash table to store the information, which includes not only the point pair length of model point cloud but also the location topology information as will be shown in section A. And in section B, the process of finding the corresponding tetrahedron will be introduced.

A. Building the two-level index hash table

Since the principle of finding two congruent tetrahedrons is to find corresponding sides with equal length, we use a linear hash function to hash the model point cloud into the corresponding buckets, as given in Equation (2).

(2) $$\begin{array}{c} \left\{\begin{array}{c} k=H\left(L\right)=floor\left(\frac{L-Lower}{\Delta l}\right)\\ \Delta l=\frac{Upper-Lower}{binNum} \end{array}\right. \end{array}$$

where, *L* is the Euclidean distance of model point pair $\left(m_{i},m_{j}\right)$, $L={∥m_{i}-m_{j}∥}_{2}$, i=1,2,…,N, j=1,2,…,N, i≠j, *N* is the number of model points. $Upper\geq L_{max}$, $L_{max}$ is the maximum Euclidean distance between two points of model point cloud. $Lower\leq L_{min}$, $L_{min}$ is the minimum Euclidean distance between two points of model point cloud. binNum is the number of buckets. *k* is the hash value, k=0,…,binNum−1.

**Figure 4:**
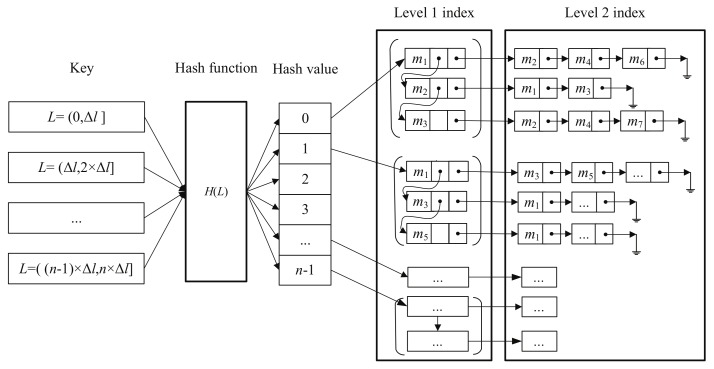
Building the two-level index hash table.

With the Equation (2), all the point pairs, with the distance in the interval [k×Δl,(k+1)×Δl] are stored in the kth bucket. In the kth bucket, the first element and the second element of point pairs are stored in level 1 index, and level 2 index, respectively. The process of building the hash table is shown in Figure 4.

B. Searching the congruent tetrahedron   

Assuming $T_{s}$ is the tetrahedron founded in scanning point cloud with vertexes $s_{0}$, $s_{1}$, $s_{2}$, $s_{3}$. HT is the two-level index hash table built with model points. The process of searching the congruent tetrahedron $T_{m}$ with vertexes $m_{0}$, $m_{1}$, $m_{2}$, $m_{3}$ in hash table is shown in Algorithm 1.

**Algorithm 1** Searching the congruent tetrahedron**Input:**
$T_{s}=\left\{s_{0},s_{1},s_{2},s_{3}\right\}$: four vertexes of $T_{s}$; HT: hash table**Output:**
$T_{m}=\left\{m_{0},m_{1},m_{2},m_{3}\right\}$: four vertexes of $T_{m}$

1:$L_{0-1}={∥s_{0}-s_{1}∥}_{2}$, $k_{0-1}=H\left(L_{0-1}\right)$ ▹$L_{i-j}$, $k_{i-j}:$ length between $s_{i}$ and $s_{j}$, and bucket number2:search the point pair $\left(m_{0},m_{1}\right)$ in the $k_{0-1}th$ bucket;3:$L_{0-2}={∥s_{0}-s_{2}∥}_{2}$, $L_{1-2}={∥s_{1}-s_{2}∥}_{2}$, $k_{0-2}=H\left(L_{0-2}\right)$, $k_{1-2}=H\left(L_{1-2}\right)$4:search the point pair $\left(m_{0},m_{2}\right)$ in $k_{0-2}th$ bucket, and $\left(m_{1},m_{2}\right)$ in $k_{1-2}th$ bucket5:**if** there is no point $m_{2}$ was found in step 4 **then**6:  goto step 2 to find another pair7:**end if**8:$L_{0-3}={∥s_{0}-s_{3}∥}_{2}$, $L_{1-3}={∥s_{1}-s_{3}∥}_{2}$, $L_{2-3}={∥s_{2}-s_{3}∥}_{2}$, $k_{0-3}=H\left(L_{0-3}\right)$, $k_{1-3}=H\left(L_{1-3}\right)$, $k_{2-3}=H\left(L_{2-3}\right)$9:search the point pair $\left(m_{0},m_{3}\right)$ in $k_{0-3}th$ bucket, the point pair $\left(m_{1},m_{3}\right)$ in $k_{1-3}th$ bucket, and $\left(m_{2},m_{3}\right)$ in $k_{2-3}th$ bucket10:**if** there is no point $m_{3}$ was found in step 9 **then**11:  goto step 4 to find another pair12:**else**13:  **return** the four points $m_{0}$, $m_{1}$, $m_{2}$ and $m_{3}$14:**end if**


#### 3.1.2. Selection Tetrahedron from Scanning Point Cloud

The farthest pair of points are extreme points on convex hull. Intuitively, the larger the volume of the tetrahedron is, the stronger the resolution is. Therefore, the selection of tetrahedron is carried out according to the principle of maximum volume. The vertexes of the tetrahedron with the largest volume must be the vertexes of the convex hull. Hence, the convex hull of scan point cloud is first constructed.

The methods of establishing convex hull include Quickhull Algorithm [34,35], Gift Wrapping Algorithm [36], Random Incremental Method [37] and so on. For the sake of time, we choose the Quickhull Algorithm with faster speed.

#### 3.1.3. Calculation of Transformation

From the set of corresponding points $s_{i}$ and $m_{i},\left(i=0,1,2,3\right)$, the transformation matrix $T_{sm}$, containing rotation matrix $R_{sm}$ and translation vector $t_{sm}$, can be calculated by Equations (3) and (4) respectively.

(3) $$\left\{\begin{array}{c} t_{sm}=O_{s}-O_{m}\\ O_{s}=\frac{\sum_{i=0}^{3}s_{i}}{4}\\ O_{m}=\frac{\sum_{i=0}^{3}m_{i}^{\prime }}{4} \end{array}\right.$$

(4) $$\begin{array}{c} \left\{\begin{array}{c} R_{sm}=\left[\begin{array}{ccc} S_{x}\cdot M_{x} & S_{x}\cdot M_{y} & S_{x}\cdot M_{z}\\ S_{y}\cdot M_{x} & S_{y}\cdot M_{y} & S_{y}\cdot M_{z}\\ S_{z}\cdot M_{x} & S_{z}\cdot M_{y} & S_{z}\cdot M_{z} \end{array}\right]\\ S_{x}=\frac{s_{1}-s_{0}}{{∥s_{1}-s_{0}∥}_{2}},S_{y}=S_{x}\times \frac{s_{2}-s_{0}}{{∥s_{2}-s_{0}∥}_{2}},S_{z}=S_{x}\times S_{y}\\ M_{x}=\frac{m_{1}-m_{0}}{{∥m_{1}-m_{0}∥}_{2}},M_{y}=M_{x}\times \frac{m_{2}-m_{0}}{{∥m_{2}-m_{0}∥}_{2}},M_{z}=M_{x}\times M_{y} \end{array}\right. \end{array}$$

#### 3.1.4. CTA Failure Detection Approach

**Figure 5:**
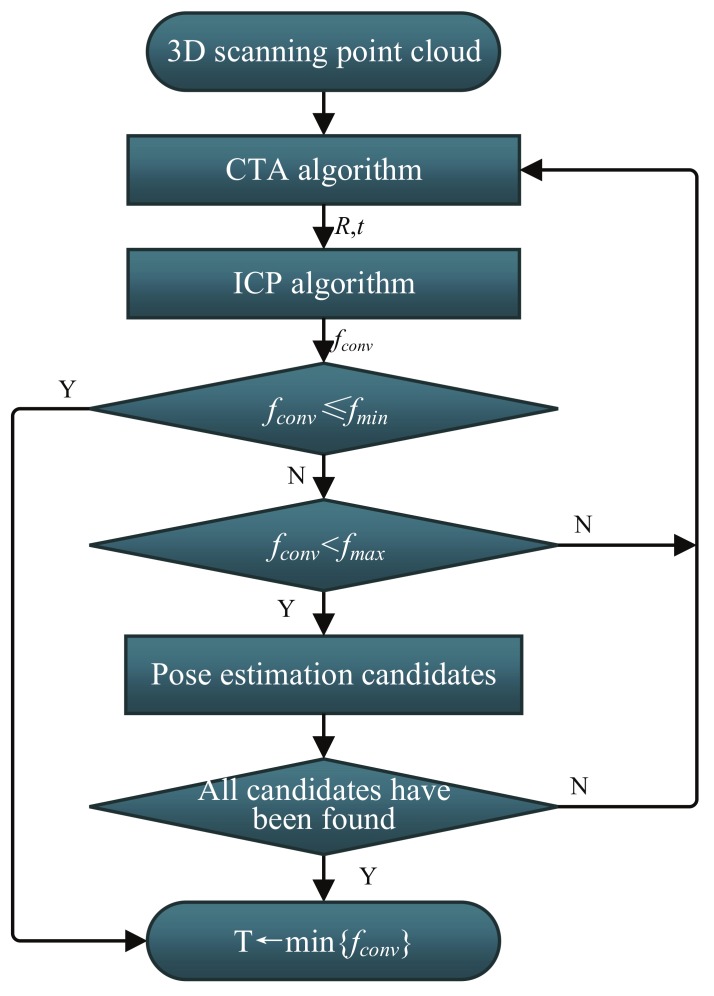
Logic of successful pose estimation for the CTA algorithms.

In general, more than one corresponding tetrahedron can be found in the hash table, but only one is correct. In order to deal with this case and choose the correct one, a strategy is used to detect the failure of the CTA algorithm autonomously. The logic of the approach is shown in Figure 5. When a corresponding tetrahedron is found, the relative transformation is calculated as described in Section 3.1.3 and the transformation is applied to the whole scanning point cloud. After that, the ICP algorithm is used to refine the registration result. The accuracy measurement of the algorithm is ICP convergence score $f_{conv}$, the minimum value of the closest point to the Euclidean distance which is calculated by Equation (5). Moreover, two threshold values $f_{max}$ and $f_{min}$ are set up. If $f_{conv}\leq f_{min}$, the registration is believed to be correct, then it is used as the input parameters of the pose tracking stage. Otherwise, if $\left(f_{conv}>f_{min}\right)$ & $\left(f_{conv}<f_{max}\right)$, it is saved as a candidate, until all of the candidates are looked up in the hash table. After that, choose the transformation corresponding to the smallest $f_{conv}$ as the output of the CTA for the input of the pose tracking.

(5) $$\begin{array}{c} f_{conv}=\frac{1}{n}\sum_{i=1}^{n}{∥m_{i}-\left(Rs_{i}+t\right)∥}^{2} \end{array}$$

### 3.2. Pose Tracking

The output of CTA will be used as the input of pose tracking and the ICP algorithm is used for pose tracking. Although the CTA algorithm has a high success rate, which has been verified in Section 5, a stability strategy is added in order to further guarantee the correctness of the initial pose. The strategy is shown as follows:

(1) For the first scanning point cloud, using CTA algorithm to calculate the transformation $T_{sm1}$;
(2) For the second frame, using CTA algorithm to calculate the transformation $T_{sm2}$;
(3) For the second frame, calculating the transformation ${T_{sm2}}^{{}^{\prime }}$ using $T_{sm1}$ as the initial pose in another thread;
(4) Computing the relative translation between $T_{sm2}$ and ${T_{sm2}}^{{}^{\prime }}$, $\Delta T={T_{sm2}}^{-1}{T_{sm2}}^{{}^{\prime }}$, and the Euler angles Δα, Δβ, Δγ can be determined from ΔT. If $\left(\Delta \alpha \leq e_{\alpha }\right)\&\left(\Delta \beta \leq e_{\beta }\right)\&\left(\Delta \gamma \leq e_{\gamma }\right)$ ($e_{\alpha },e_{\beta },e_{\gamma }$ are the thresholds), the CTA algorithm is considered as a stable and accurate initial value, and then goto step (5). Otherwise, the result of the CTA algorithm is incorrect, then replace $T_{sm2}$ with ${T_{sm2}}^{{}^{\prime }}$ and go to step (3);
(5) For the subsequent frames, using ICP algorithm to calculate the transformation $T_{smi}$ for pose tracking.

## 4. Analysis of Influence Factors

**Figure 6:**
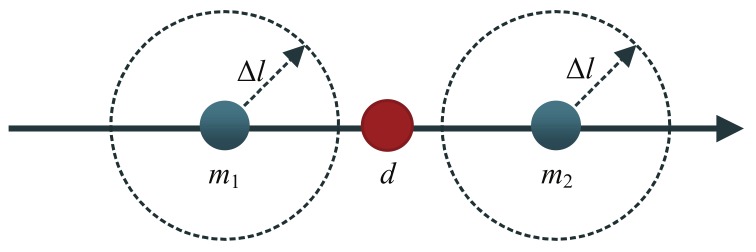
A small Δl incurring a failure of finding a corresponding point.

According to hash function (Equation (2)) and the process of searching in the hash table, the main influencing factors are the number of buckets binNum which is equivalent to Δl, and the number of point pairs in each bucket which is related to the distance interval of the model point cloud $I_{v}$. As shown in Figure 6, $m_{1}$ and $m_{2}$ are points of model point cloud, *d* is one of the ideal corresponding points in $T_{m}$, $m_{1}$ and $m_{2}$ are adjacent to *d*. If $\left|m_{2}-m_{1}\right|>\Delta l$, then it could not guarantee that *d* will fall into the bucket that contains $m_{1}$ or/and $m_{2}$. This means that it may not be possible to find the correct match in the hash table. Only when $\left|m_{2}-m_{1}\right|\leq \Delta l$, it can be ensured that no matter how the distance grid around *d* is divided, *d* must be in the bucket that contains $m_{1}$ or/and $m_{2}$.

**Figure 7:**
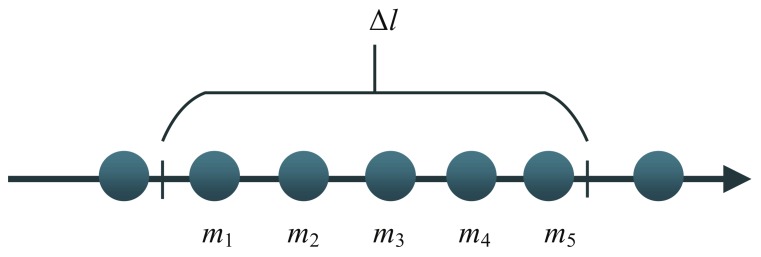
A large Δl introducing redundant candidates.

Taking account of all the point pairs of model points, it can be ensured that the match candidates contain the global optimal matching, as long as $\Delta l\geq I_{v_{sparse}}$ ($I_{v_{sparse}}$ is the largest distance interval of the model point cloud). However, if $I_{v_{sparse}}$ is much larger than the other interval, more points will be hashed in the same bucket as shown in Figure 7. Assuming that $m_{3}$ is the correct model point corresponding to *s*, a vertex of $T_{s}$. All of the sides containing $m_{3}$ will be retrieved as equally as possible to replace $m_{3}$ with $m_{1}$,$m_{2}$,$m_{4}$ and $m_{5}$. Therefore, it is easy to bring redundant matches to increase Δl. Simply, if $I_{v_{sparse}}=I_{v_{dense}}$ ($I_{v_{dense}}$ is the smallest distance interval of the model point cloud), we can get a balance between time and accuracy. Therefore, the model point cloud be sampled uniformly at a interval of $I_{v}$ , and let $\Delta l=I_{v}$.

## 5. Experiments

**Figure 8:**
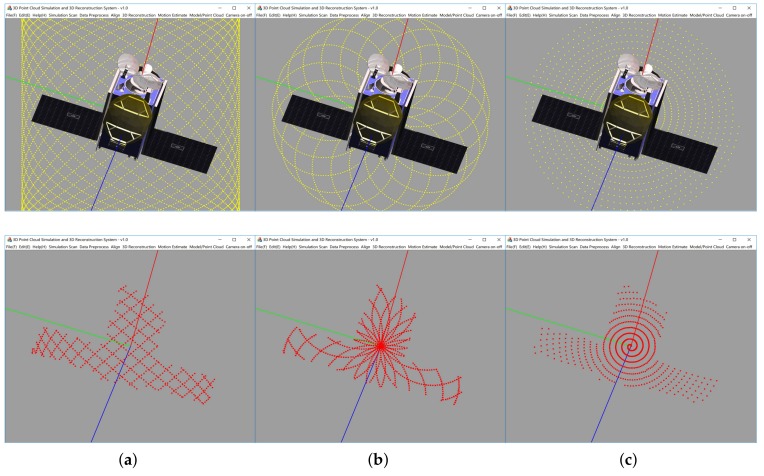
The 3D point cloud simulation software (**a**) Lissajous, (**b**) Rosette, (**c**) Spiral.

In this section, we have performed numerical simulation experiments and a field experiment to test the performance of the algorithm. In the experiments, the target is a 1:6 scale satellite model as shown in Figure 8. The size of scale target model is 1521mm×559mm×870mm. Simulations are carried out in C++ environment run by a commercial desktop PC equipped with an Intel${}^{TM}$ I5 CPU at 2.4GHz.

### 5.1. Simulation System

We also developed a 3D point cloud simulation software. After entering the 3D model, and set the relevant scanning parameters, the output of the laser scanner can be simulated. 3D point cloud simulation software is as shown in Figure 8:

In the simulation system, the three scanning patterns of Lissajous, Rosette and Spiral, all of which can cover an area in the fastest time [38], are implemented. The Rosette and Spiral patterns have a high density of points in the middle of the field of view, which will increase the number of points and lead to a more time-consuming process of constructing the 3D convex hull. By contrast, the Lissajous pattern can obtain more uniform points in the middle of the field of view. Therefore, the Lissajous pattern is better in filed experiments. In order to ensure the consistency of the simulation and field experiments, all experiments were conducted using the Lissajous pattern.

### 5.2. Impact of Different binNum

According to the discussion in Section 4, the point cloud of model should be uniform. We use the software of Geomagic Studio to convert the 3D geometric model to point cloud, and further reduce the number of points uniformly to 484 with the interval $I_{v}$ = (348 mm∼521 mm). The two parameters Δl and $I_{v}$ involved in the algorithm are interrelated through the analysis. The impact of Δl on the CTA algorithm and the relative between Δl and $I_{v}$ are verified in the case of fixed $I_{v}$ obtained above.

Through Equation (2), Δl and binNum are equivalent. Therefore, in this section we discuss the efficiency of different binNum on the accuracy and speed of the algorithm. After the equidistant sampling of the model point cloud, the interval is about (348 mm∼521 mm). During the process of acquiring the simulated scanning point cloud, the noise with standard deviation of 200 mm is added in the direction of the ray. The average estimation pose errors of different binNum are shown in Table 1. All of the Euler angles in the experiments use the Z-X-Y convention.

According to Equation (2), binNum = 28∼19 under the $I_{v}$ = (348 mm∼521 mm). From the results in Table 1, it is obvious that with the increase of the number of buckets, the registration time gradually decreases and the registration error rate increases. When the binNum is between 20 and 30, better registration accuracy and lower error rate are achieved, and the registration time is less than 100 ms. This conclusion is consistent with the discussion in Section 4.

In order to balance the registration time and the registration accuracy, binNum=25 is taken in the following experiments.

### 5.3. CTA Algorithm Test

**Figure 9:**
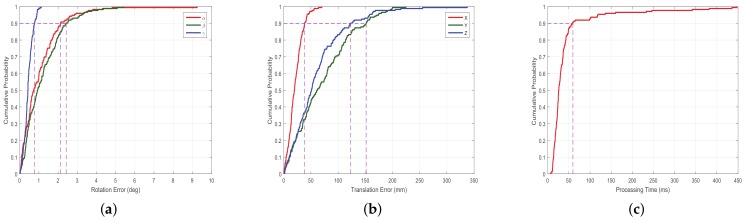
Performance of congruent tetrahedron align algorithm. (**a**) CDF of rotation error; (**b**) CDF of translation error; (**c**) CDF of processing time.

The accuracy of pose tracking is closely related to the initial attitude estimation results, so it is necessary to test the performance of initial attitude estimation algorithm CTA. In this simulation experiment, we also added the noise with standard deviation of 200 mm in the direction of ray and selected 10 groups of 20 positions for testing. The absolute error of initial translation error, initial rotation error and time consuming are given in Figure 9.

Figure 9 shows the Cumulative Distribution Function (CDF), we can see that the proposed relative pose estimation method can estimate the real-time relative pose effectively. The 90% translation error is less than 150 mm, and 90% rotation error is less than $2.5^{\circ }$. Furthermore, we can see that 90% time-consuming is less than 70 ms. The time-consuming and accuracy of transformation satisfy pose tracking.

### 5.4. Pose Tracking Test

To test the efficiency of the proposed method for pose tracking, simulated experiments for tumbling targets, which exist widely in space, are carried out under the following different conditions.

**Figure 10:**
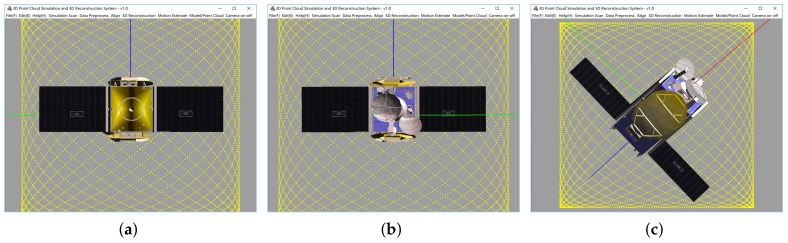
Three different initial attitudes of numerical simulated experiments. (**a**) $\alpha =0^{\circ },\beta =0^{\circ },\gamma =0^{\circ }$; (**b**) $\alpha =0^{\circ },\beta =180^{\circ },\gamma =0^{\circ }$; (**c**) $\alpha =-120^{\circ },\beta =0^{\circ },\gamma =40^{\circ }$.

In test 1, the initial attitude (α,β,γ) is set to $\left(0^{\circ },0^{\circ },0^{\circ }\right)$, as shown in Figure 10a, and the target rotates around the model’s Y-axis (blue axis). For each simulated point cloud, the β is changing from $0^{\circ }$ to $360^{\circ }$ at $2^{\circ }$ interval;

In test 2, the initial attitude (α,β,γ) is set to $\left(0^{\circ },0^{\circ },0^{\circ }\right)$, as shown in Figure 10a, and the target rotates around the model’s Z-axis (axis perpendicular to the solar array of the model and perpendicular to the screen). For each simulated point cloud, the γ is changing from $0^{\circ }$ to $180^{\circ }$ at $2^{\circ }$ interval;

In test 3, the initial attitude (α,β,γ) is set to $\left(0^{\circ },180^{\circ },0^{\circ }\right)$, as shown in Figure 10b, and the target rotates around the model’s Z-axis (axis perpendicular to the solar array of the model and perpendicular to the screen). For each simulated point cloud, the γ is changing from $0^{\circ }$ to $180^{\circ }$ at $2^{\circ }$ interval;

In test 4, the initial attitude (α,β,γ) is set to $\left(-120^{\circ },0^{\circ },40^{\circ }\right)$, as shown in Figure 10c, and the target rotates around the model’s Z-axis (axis perpendicular to the solar array of the model).

**Figure 11:**
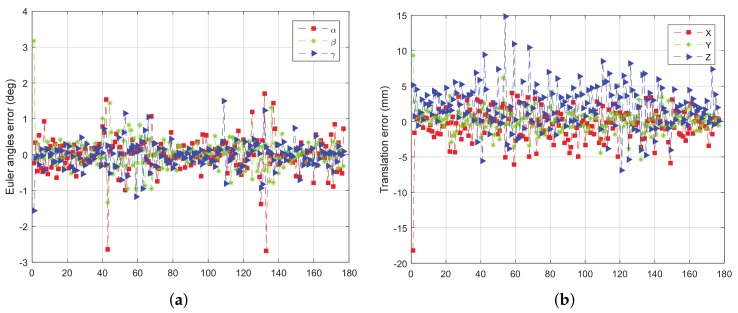
The error curve of (**a**) rotation and (**b**) translation of test 1.

**Figure 12:**
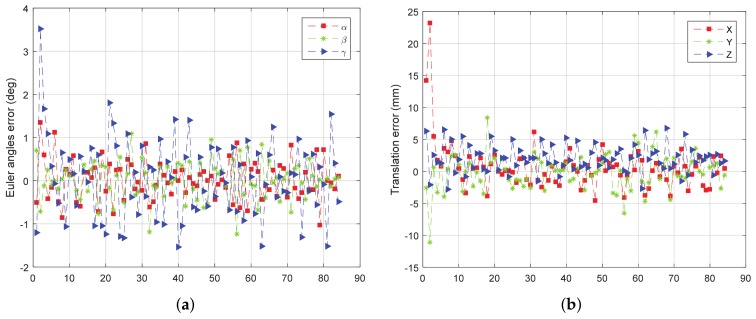
The error curve of (**a**) rotation and (**b**) translation of test 2.

**Figure 13:**
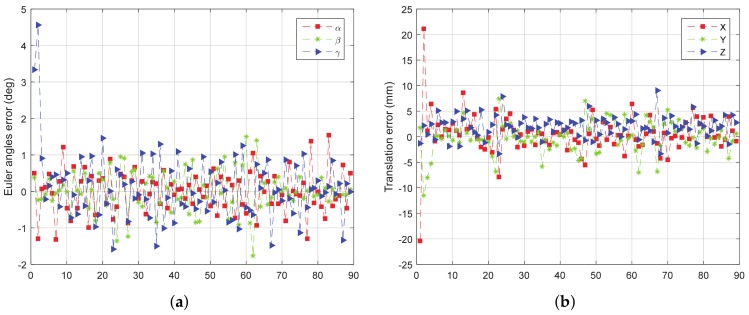
The error curve of (**a**) rotation and (**b**) translation of test 3.

**Figure 14:**
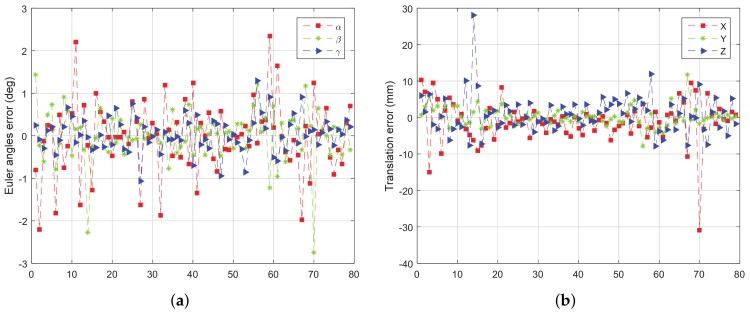
The error curve of (**a**) rotation and (**b**) translation of test 4.

When simulating the LIDAR to get point cloud, the white noise with a standard deviation of 200mm is added in the direction of the simulated rays. The rotation error and translation error of the four tests are given from Figure 11, Figure 12, Figure 13 and Figure 14.

From Figure 11, Figure 12, Figure 13 and Figure 14, we can see that, the rotation error is almost less than $1^{\circ }$, and the translation error is almost less than 10 mm in different initial attitudes and different rotation axes. This indicates that the CTA computing initial position proposed in this paper, and pose tracking by ICP algorithm is accurate, fast and robust. However, there also exist some outliers which can be divided into two groups. One of the groups appears in the first few frames, which is due to the large error of the initial pose estimation. The other group appears in the frames, e.g., frame 43 and 133 in Figure 11a, in which the LIDAR can only scan a small part of the target. Under this condition, the feature of the scanning point cloud is not obvious. At the same time, the standard ICP algorithm tends to fall into a local optimal solution, leading to an increase in the error.

### 5.5. Field Experiment

**Figure 15:**
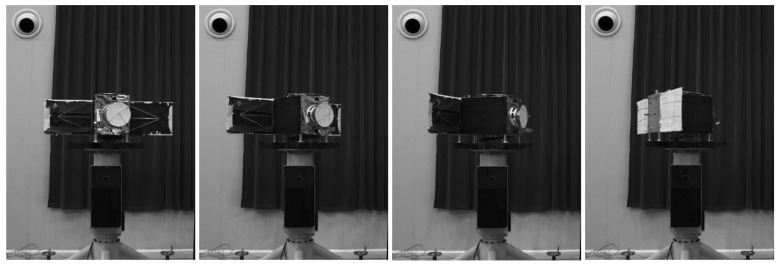
Field experiment.

In order to test the real performance of the algorithm, a field experiment was carried out. The satellite model with a 1:6 scale, shown in Figure 15, was fixed on a 6-DOF turntable, and the model can follow the turntable to make a rotary motion about the fixed axis. The control precision of the turntable is $0.01^{\circ }$.

The measurement sensor is a single-line scanning 3D-LIDAR (Time-of-Flight) system developed by BICE and the point cloud acquisition frequency is greater than 10 Hz.

**Figure 16:**
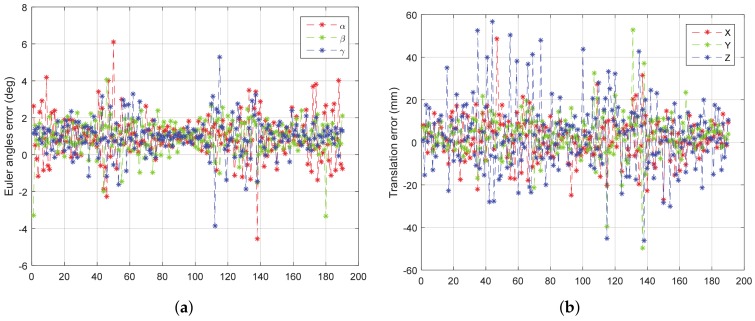
The error curve of (**a**) rotation and (**b**) translation of field experiment.

When the target rotates continuously, the points outside the target cannot be easily removed. Therefore, only the experiment about rotating around the y-axis is performed. Based on known information such as the distance between the target and the wall, we can filter out the noise outside the model that does not appear in space. Turntable rotation speed is set to be $10^{\circ }$/*s*, the frequency of point cloud data acquisition is set to be 10 Hz. In this experiment, the target only rotates around the y-axis without translation. The experimental results are as shown in Figure 16.

From Figure 16, we can see that both the rotation error and the translation error of the field experiment are increased compared with the results of simulation experiments. The reason is that the measurement error of LIDAR itself is more complicated than the simulation error, and the rotation of the target also leads to the deformation of the point cloud data. However, the overall result still shows good accuracy and stability; the rotation error is almost less than $2^{\circ }$, and the translation error is almost less than 20 mm.

## 6. Conclusions

In this paper, a high accuracy, good real-time and reliable initial pose determination method CTA, which can be used for sparse unordered LIDAR data, is proposed to provide a reliable initial value for tracking. Based on the known target model, this method uses the congruent tetrahedron approach to estimate relative pose, and then uses ICP algorithm for pose tracking. The two main parameters that affect the CTA algorithm are analyzed in detail. The 3D target model point cloud should be resampled as uniform as possible, and there is a speed/accuracy tradeoff when the Δl value nears to the point cloud interval value which have been verified in the subsequent simulation experiments. At the same time, the numerical simulated experiments are completed in a variety of attitudes by artificially adding noise. The results denote that the proposed CTA algorithm is accurate, efficient and robust. The results of numerical simulated experiments and field experiments, which simulate the motion of a high-speed spinning target, demonstrate that the proposed method CTA to estimate the Initial pose and ICP method to track pose can satisfy the real-time calculation of 6-DOF pose of high-speed tumbling target system.

In addition, the LIDAR point cloud in this algorithm can be very sparse and unorganized. The method also can be used for dense point cloud only if the down sampling is used to reduce the time off establishing the convex hull, to ensure the real-time performance of the algorithm. Therefore, it is suitable for other types of LIDAR systems, such as flash LIDAR.

In future work, the robustness of the CTA algorithm will be analyzed considering the different shape and various kinematic states of the targets. In addition, the situation in which the target is occluded also needs to be considered in future work.

## Figures and Tables

**Table 1:** The average estimation pose error of different binNum.

*binNum*	Time/ms	Points Num	$\alpha {/}^{\circ }$	$\beta {/}^{\circ }$	$\gamma {/}^{\circ }$	*X*/mm	*Y*/mm	*Z*/mm	Error Rate/%
5	28,752.59	102	1.36	1.60	0.50	23.53	81.40	81.82	0
10	1703.99	102	1.53	1.58	0.52	25.04	75.07	85.24	0
15	221.18	102	1.92	1.63	0.51	27.00	70.12	92.00	0
20	62.24	102	1.76	1.92	0.51	27.16	75.03	85.45	0
25	33.27	102	1.77	1.55	0.54	24.98	86.77	81.04	2.8
30	17.23	102	1.72	1.77	0.53	25.63	75.01	83.10	5.7
35	17.17	102	1.50	1.78	0.53	22.45	85.64	95.75	11.4
40	15.11	102	1.30	1.46	0.49	21.34	96.88	72.48	25.7
50	14.98	103	1.46	1.41	0.56	22.79	88.69	89.51	34.3
60	13.14	104	1.60	1.52	0.37	19.16	69.69	71.50	60
70	14.49	106	2.78	2.01	0.55	23.17	94.40	60.70	85.7
80	13.95	105	2.03	1.58	0.52	26.64	89.31	76.10	80
